# Antenatal Ultrasonographic Diagnosis of a Constellation of Alobar Holoprosencephaly, Ethmocephaly, and Hydronephrosis in a Case of Early-Onset Intrauterine Growth Retardation: A Case Report

**DOI:** 10.7759/cureus.27375

**Published:** 2022-07-27

**Authors:** Prasanthi R Ghanta, Suresh Phatak, Pratik J Bhansali, Bhavik S Unadkat, Nidhi Goyal

**Affiliations:** 1 Radiodiagnosis, Datta Meghe Institute of Medical Sciences, Wardha, IND; 2 Obstetrics and Gynecology, Datta Meghe Institute of Medical Sciences, Wardha, IND

**Keywords:** intrauterine growth retardation, ultrasonography, hydronephrosis, ethmocephaly, alobar holoprosencephaly

## Abstract

Alobar holoprosencephaly is a congenital malformation that results from failure of the forebrain/prosencephalon to divide into right and left halves. Despite the literature on the genetic and chromosomal abnormalities associated with this condition, information on additional causes and explanations for variability in phenotypic expressivity are lacking. We report a case of early-onset intrauterine growth retardation with alobar holoprosencephaly, ethmocephaly, and hydronephrosis diagnosed on antenatal ultrasonography in a 27-year-old primigravida with no known risk factors or family history. The combination of holoprosencephaly with associated midline facial anomalies and the genitourinary abnormality, in this case, constitutes a rare phenotypic presentation. This case emphasizes the importance of antenatal ultrasonography in the early detection of lethal anomalies like alobar holoprosencephaly. The pregnancy was safely terminated in accordance with the mother’s decision.

## Introduction

Holoprosencephaly (HPE) is a rare congenital malformation but one of the most common among central nervous system anomalies, with an incidence of 1:16,000 at birth and 1:250 among the conceptuses [1].

HPE spectrum has distinct entities like alobar, semi-lobar, or lobar HPE [2], milder variant called syntelencephaly/midline interhemispheric variant (MIH); micro and minimal forms. Among these, alobar HPE is the most severe and fatal form. HPE can be associated with midline facial malformations [3] such as ethmocephaly (hypotelorism and proboscis), cleft lip and palate, single midline maxillary incisor, and depressed nasal bridge.

Embryologically, three primary vesicles are formed after the process of primary neurulation is completed. They are prosencephalon, mesencephalon, and rhombencephalon. The prosencephalon is formed by ventral induction and involves three processes, namely, formation, division/cleavage, and midline development, which occur in the same sequential order. Disorders of the cleavage process in the fifth week of gestation result in the HPE spectrum. Failure of the eye field to divide under the signaling influence of the prechordal plate can result in cyclopia [4].

Unilateral fetal hydronephrosis is most commonly caused by pelvic-ureteric junction (PUJ) obstruction, which has multifactorial causation. Anomalies involving the urinary system are one of the most common and can frequently occur as part of different genetic syndromes or sporadic malformations. Development of the urinary tract begins in the third week of embryonic life. Three intermediate mesoderm structures, namely, metanephric blastema, ureteric bud, and glomerular capillary network, interact together to form the permanent kidney under the influence of a variety of molecular signals. The upper urinary tract including the pelvicalyceal system is derived from the ureteric bud [5].

HPE can occur in conjunction with other system anomalies, including the urinary system, as depicted in the present case.

## Case presentation

A 27-year-old primigravida female with no known comorbidities, maternal risk factors, or any significant family history (like consanguineous marriage or congenital anomaly in first- or second-degree relatives) came for antenatal ultrasonography (USG) at 20 weeks of gestational age (by last menstrual period and dating scan). She had no history of exposure to harmful medications, infections, toxins, radiation, or any other adverse events. The patient did not have prior antenatal USG scans and was not on prenatal vitamins/folic acid supplementation in the pre-conceptional or pregnancy period.

**Figure 1:**
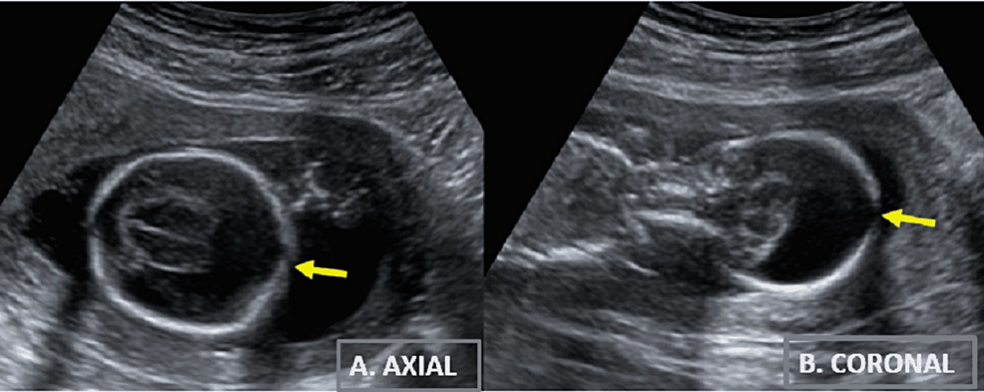
B-mode grayscale two-dimensional USG images (A) Axial and (B) coronal sections of fetal head demonstrating mono ventricle with the absence of falx and fused thalami.

On antenatal USG examination, the average gestational age of the fetus was 16 weeks and six days with an estimated fetal weight of 166 grams (<3rd centile according to gestational age by her dating scan), suggesting early-onset intrauterine fetal growth restriction. On USG, different anomalies were found in the developing fetus. The forebrain of the fetus appeared to have anechoic fluid predominantly, with no discernible falx, forming a mono/holoventricle with a very thin rim of brain tissue peripherally (Figure 1). Midline structures such as the corpus callosum, cavum septum pellucidum, and the third ventricle were not visualized. The thalami were fused in the midline.

**Figure 2:**
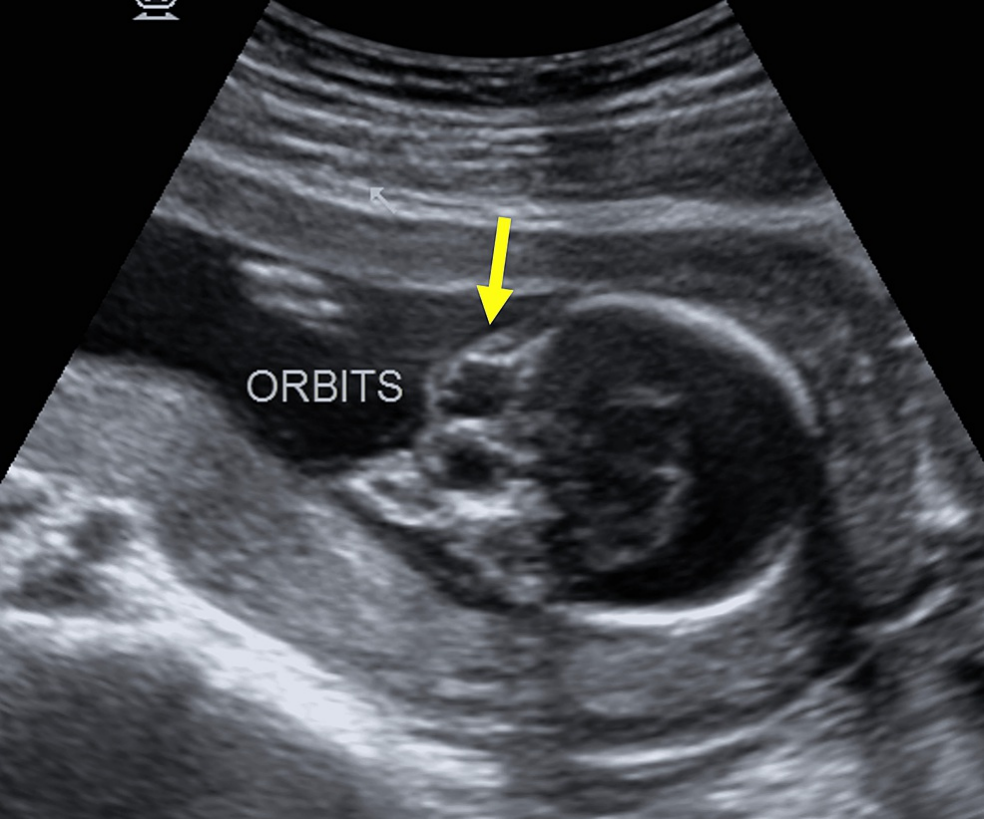
B-mode grayscale two-dimensional USG image Image of the fetal orbits showing hypotelorism.

**Figure 3:**
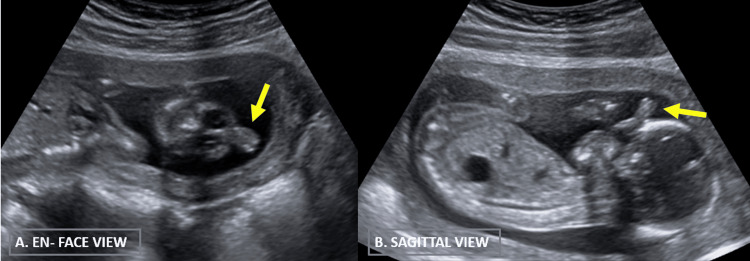
B-mode grayscale two-dimensional USG images (A) En face view and (B) sagittal view of the fetal face demonstrating midline soft tissue projection from the forehead suggestive of proboscis.

En face view and sagittal view showed midline facial anomalies like ethmocephaly (comprising of hypotelorism as shown in Figure 2 and proboscis as shown in Figure 3).

**Figure 4:**
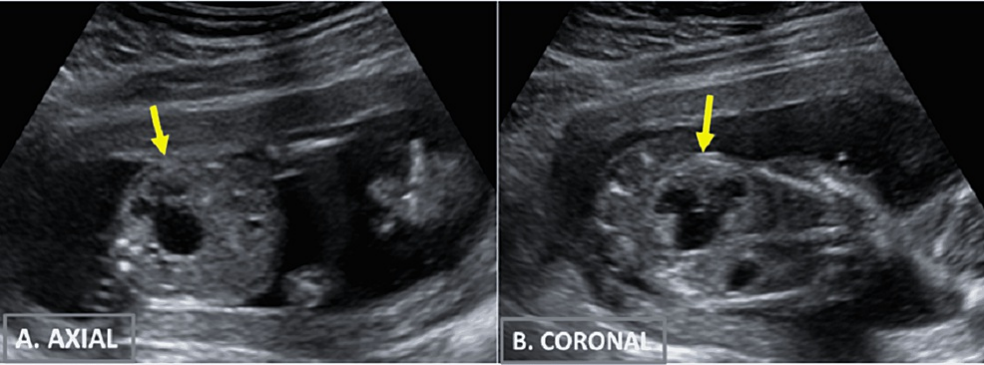
B-mode grayscale two-dimensional USG images (A) Axial and (B) coronal sections of the fetal abdomen demonstrating gross dilatation of the pelvicalyceal system of the right kidney suggestive of hydronephrosis.

There was also the presence of gross dilatation of the renal pelvis and the calyces on the right side (anteroposterior diameter of the pelvis was 10 mm) with thinning of renal parenchyma and poor corticomedullary differentiation (Figure 4). The left kidney and urinary bladder were normal (bladder longitudinal diameter was 8 mm). The rest of the examination of the fetus, including the heart, spine, limbs, etc., was normal.

The patient was referred to an obstetrician. After counseling of the patient and her spouse regarding the prognosis and management options, the patient expressed her decision to terminate the pregnancy.

On blood investigations, the patient was found to be hepatitis B Ag positive. The other blood investigations and physical examinations were found to be normal.

**Figure 5:**
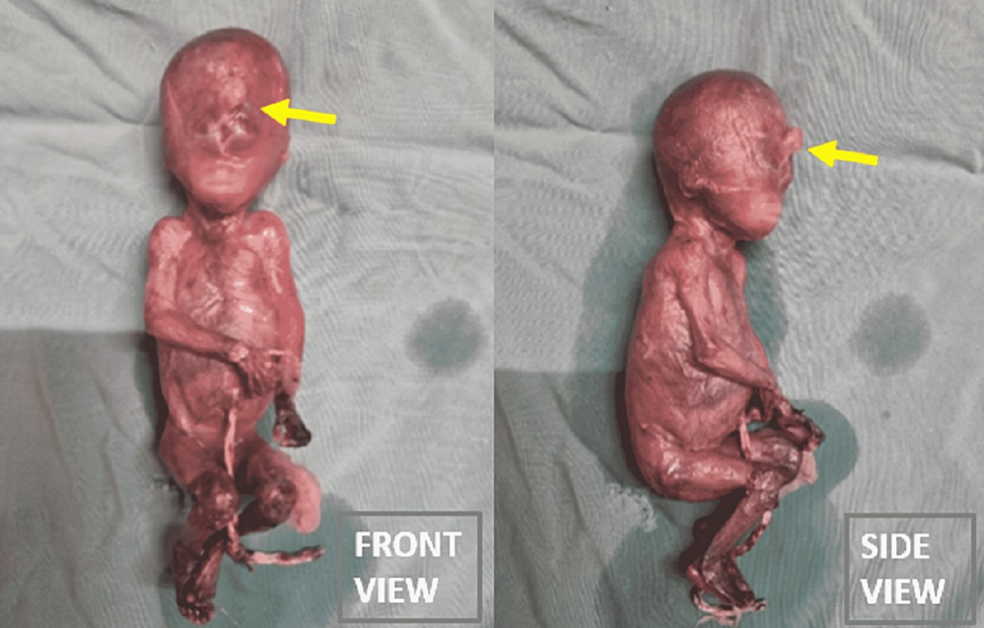
Gross specimen of the abortus (A) Front view and (B) side view of the abortus showing midline facial anomalies of ethmocephaly (hypotelorism and proboscis in between the rudimentary eyes). The limbs appear grossly normal.

The possibility of syndromic association was hypothesized to be the cause of the constellation of HPE, midline facial defects, and unilateral hydronephrosis diagnosed in this case. The patient was offered a histopathological examination and genetic study of the abortus (Figure 5). However, the family was unwilling for the investigation of abortus because of socio-cultural reasons. The pregnancy was terminated respecting the mother’s decision. The mother had an uneventful recovery after the termination of pregnancy.

## Discussion

The risk factors for HPE include maternal diabetes mellitus, exposure to alcohol, cigarette smoking, certain medications, retinoic acid, isotretinoin, methotrexate, etc. [6].

The patterns of inheritance are varied. HPE can occur as an isolated malformation or often as a part of various syndromes that have non-central nervous system congenital anomalies in addition to HPE. Various chromosomal anomalies, including aneuploidy (trisomy 13 and 18), triploidy, deletion syndromes, and mutations of different genetic loci (e.g., those associated with Sonic Hedgehog signaling and cholesterol biosynthesis pathways) [7] have been described in the literature to be associated with this disorder. 13q deletion syndrome can manifest with congenital malformations of the eyes, face, kidneys/urinary tract, and heart, in addition to HPE [8].

Babies born with HPE variants invariably have high morbidity with central nervous system manifestations such as epilepsy, developmental delay, endocrine dysfunction, risk of poor nutrition, aspiration, and homeostasis imbalance [9]. High mortality is seen in more severe forms in the first month of life.

Early prenatal diagnosis of alobar HPE allows the mother to choose safe termination of pregnancy before 20 weeks of gestational age as such gross anomalies can be diagnosed as early as the late first trimester.

The antenatal USG has excellent sensitivity and specificity in diagnosing HPE. The imaging features of alobar HPE are absent falx, forebrain that is replaced largely by cerebrospinal fluid (holoventricle) with the thin peripheral mantle of brain parenchyma, absent midline structures like cavum septum pellucidum, corpus callosum, and third ventricle, and the presence of fused thalami in the midline.

Semi-lobar HPE is distinguished from the alobar variety by the presence of rudimentary interhemispheric fissure, posterior horns of lateral ventricles, anterior part of the corpus callosum, rudimentary cavum septum pellucidum, small third ventricle, etc., which represent the incomplete separation of the prosencephalon.

Hydranencephaly and severe hydrocephalus are differentiated from alobar HPE by the presence of falx, non-fusion of thalami, and absence of associated midline facial anomalies [10].

Hypotelorism is defined as the interorbital diameter < 5th percentile for gestational age. It is a part of the spectrum of midline migration defects associated with HPE. Extreme hypotelorism can result in cyclopia [4,11].

Unilateral obstructive uropathy is one of the most common congenital anomalies that can occur isolated or as a part of a constellation of anomalies in various genetic syndromes. Hydronephrosis has gestational age-specific criteria for the diagnosis with an anteroposterior diameter of the renal pelvis ≥ 4 mm denoting mild hydronephrosis and ≥7 mm or associated calyceal dilatation denoting moderate/severe hydronephrosis in the second trimester [12], as seen in this case.

The presence of any congenital anomaly should warrant careful scrutiny to rule out the presence of associated anomalies involving other organ systems. Genetic studies are undertaken at the institutions where they are available to determine any genetic cause of anomalies, and the involved couples are provided genetic counseling for planning future pregnancies [13].

## Conclusions

This case report underscores the importance of USG in the timely antenatal diagnosis of lethal congenital anomalies in resource-poor settings such as rural India. While advanced imaging modalities like MRI are sometimes used to confirm central nervous system anomalies in doubtful cases, they are either inaccessible or expensive in the rural Indian setting. Early ultrasonographic diagnosis in experienced hands can avoid the high mortality and morbidity associated with lethal anomalies and allow the mother to make an informed choice to safely terminate a pregnancy at the earliest stage possible.
